# Bone scintigraphy based on deep learning model and modified growth optimizer

**DOI:** 10.1038/s41598-024-73991-8

**Published:** 2024-10-27

**Authors:** Omnia Magdy, Mohamed Abd Elaziz, Abdelghani Dahou, Ahmed A. Ewees, Ahmed Elgarayhi, Mohammed Sallah

**Affiliations:** 1 Applied Mathematical Physics Research Group, Physics Department, Faculty of Science, Mansoura University, Mansoura, 35516 Egypt; 2 Present Address: Department of Mathematics, Faculty of Science, Zagazig University, Zagazig, 44519 Egypt; 3 Faculty of Computer Science and Engineering, Galala University, Suze, 435611 Egypt; 4 Artificial Intelligence Research Center (AIRC), Ajman University, Ajman, 346 United Arab Emirates; 5 Mathematics and Computer Science department, University of Ahmed DRAIA, Adrar, 01000 Algeria; 6 Present Address: School of Computer Science and Technology, Zhejiang Normal University, Jinhua, 321004 China; 7 Department of Information System, College of Computing and Information Technology, University of Bisha, P.O Box 551, Bisha, 61922 Saudi Arabia; 8 Department of Physics, College of Sciences, University of Bisha, P.O. Box 344, Bisha, 61922 Saudi Arabia

**Keywords:** Bone scintigraphy, Bone metastasis, Growth optimizer (GO), Arithmetic optimization algorithm (AOA), Nuclear medicine, Computational biology and bioinformatics, Image processing, Machine learning

## Abstract

Bone scintigraphy is recognized as an efficient diagnostic method for whole-body screening for bone metastases. At the moment, whole-body bone scan image analysis is primarily dependent on manual reading by nuclear medicine doctors. However, manual analysis needs substantial experience and is both stressful and time-consuming. To address the aforementioned issues, this work proposed a machine-learning technique that uses phases to detect Bone scintigraphy. The first phase in the proposed model is the feature extraction and it was conducted based on integrating the Mobile Vision Transformer (MobileViT) model in our framework to capture highly complex representations from raw medical imagery using two primary components including ViT and lightweight CNN featuring a limited number of parameters. In addition, the second phase is named feature selection, and it is dependent on the Arithmetic Optimization Algorithm (AOA) being used to improve the Growth Optimizer (GO). We evaluate the performance of the proposed FS model, named GOAOA using a set of 18 UCI datasets. Additionally, the applicability of Bone scintigraphy for real-world application is evaluated using 2800 bone scan images (1400 normal and 1400 abnormal). The results and statistical analysis revealed that the proposed GOAOA algorithm as an FS technique outperforms the other FS algorithms employed in this study.

## Introduction

Bone metastasis is one of the most serious dangers of malignant tumors and the primary cause of mortality in cancer patients^1,2^. The most prevalent malignant bone lesion is bone metastases. 30–70% of cancer patients have skeletal involvement^3–5^. Thus, early identification of bone cancer is critical in deciding treatment decisions^6^. A bone scan is a nuclear medicine imaging screening of the bones throughout the body. It has a high sensitivity for detecting bone metastases and can detect them early^7,8^. At the moment, doctors read bone scan images manually to diagnose them; the area with a lesion in the bone scan image frequently looks like a tiny area with several lesions. Furthermore, the image quality of the bone scan is poor. Patients may miss the best therapy window, resulting in further disease development and a bad prognosis. So, predicting the onset of bone metastases is critical. As a result, using a machine learning system to detect the position and size of the lesion region can dramatically minimize (doctor.

Prior studies on machine learning applications in tumor detection and prediction include works by D’Angelo et al.^9^, Bai et al.^10^, Liu et al.^11^, Shrivastava et al.^12,13^, and Zhao et al.^14^. These studies explore various techniques such as logistic regression, support vector machine, and deep learning methods for tasks like segmentation, lesion identification, and cancer diagnosis. Despite advancements, existing models often lack the required accuracy for medical diagnosis. To address this, we propose a novel AI model based on the modification of a Growth Optimizer (GO) using the Arithmetic Optimization Algorithm (AOA). The efficacy of GO and AOA has been demonstrated in diverse applications. Fatani et al.^15^ used a modified GO for an Intrusion Detection System (IDS). Aribia et al.^16^ applied GO to estimate the characteristics of solar PV modules. Gao et al.^17^ developed a quadruple parameter adaptation growth optimizer (QAGO), while Nguyen et al.^18^ focused on optimizing power loss in electrical distribution networks. Agushaka et al.^19^ introduced nAOA, an improved variant of AOA. Chen et al.^20^ proposed the improved arithmetic optimization algorithm (IAOA), which efficiently utilizes population control. Khatir et al.^21^ suggested two-stage methods for damage detection in plate structures. Zheng et al.^22^ combined AOA and slime mold algorithm for optimization in a hybrid approach. Deepa et al.^23^ used AOA to optimize a VGG-16 architecture for Alzheimer’s disease classification, emphasizing pre-processing, feature extraction, feature selection, and classification as crucial stages in their study.

To acquire knowledge and extract strong feature representation from the raw Bone Scintigraphy images, we employed the MobileViT network as a hybrid lightweight DL Architecture incorporating both CNNs and ViTs (Vision Transformer). Thus, the CNN components detect and analyze local features in the images, such as edges and textures, crucial for identifying subtle anomalies in bone scans. Simultaneously, the ViT elements capture global contextual information, recognizing patterns and relationships across the entire image. Central to both training and extracting image features, the model processes input images resized to a consistent resolution of 244 × 244 pixels. Feature extraction occurs before the final classification layer in the modified MobileViT model, where the learned representations of each image are condensed into a 1 × 256 feature vector. This vector is then utilized in our framework’s feature selection algorithm for additional analysis and processing. The provided FS model is based on enhancing the Growth Optimizer (GO) method performance utilizing the Arithmetic Optimization Algorithm (AOA). Among the most current Meta-Heuristic algorithms, GO and AOA approaches show promising potential for effectively balancing the features of exploration and exploitation when resolving FS problems. The parallel cooperation reduces the likelihood of the two approaches being trapped at local optima and improves their capacity to balance exploration and explication, rather than relying just on one of them to solve the feature selection problem. This innovative combination, which has not been explored in existing literature, presents a unique contribution to the field and has the potential to be extended to a broader range of applications.

This study’s contribution can be summarized as follows:

1. Propose a machine learning technique to enhance the expectation of Bone scintigraphy founded on enhancing the performance of Growth Optimizer (GO) and Deep learning.
2. Apply the Mobile Vision Transformer (MobileViT) and lightweight CNN to excerpt the features from Bone scintigraphy images.
3. Propose an alternative feature selection model based on improving the efficiency of the Growth Optimizer algorithm by utilizing the operators of the Arithmetic Optimization Algorithm (AOA).
4. Evaluate the performance of the developed Bone scintigraphy detection technique using a collection of real-world collected images.

The rest of this article is organized as follows: “Related work” introduces relevant studies. In part 3, we analyze the differences between those works and our study, and in “Experimental results and discussion”, we present the essential notions of the two algorithms, GO and AOA. The proposed technique of the suggested GOAOA. Section “Conclusion and future work” presents the experimental results from test datasets. Section 6 then reports on the conclusions.

## Related work

In this part, we provide an overview of previous studies on metaheuristic algorithms and bone metastasis.

### Metahuristic algorithms

Machine learning’s application in medical image analysis has surged, employing metaheuristic algorithms. Dhal et al.^24^ provided an overview of the Arithmetic Optimization Algorithm (AOA) and its variations, emphasizing its broad applications in optimization disciplines. Heidari et al.^25^ introduced the Harris Hawks Optimizer (HHO), a nature-inspired approach, focusing on dynamic pattern optimization. Cikan et al.^26^ used the EO algorithm for power distribution network reconfiguration, providing a comprehensive comparison of common techniques. Abdulsalami et al.^27^ enhanced AOA with a differential evolution mutation technique and chaotic local search. Zhao et al.^28^ introduced the Atom Search Optimization (ASO) based on molecular dynamics for versatile optimization. Zhang et al.^29^ demonstrated CDESSA, combining differential evolution and chaotic initialization. Mirjalili et al.^30^ proposed Multiobjective Salp Swarm Algorithm (MSSA) and Salp Swarm Algorithm (SSA) inspired by salps’ swarming activity. Wang et al.^31^ reviewed Particle Swarm Optimization (PSO), examining its applications and theory. Pan et al.^32^ proposed BBFGO, a binary variant of BFGO, addressing the algorithmic slump problem. Liu et al.^33^ suggested an Improved Feature Selection approach (IFS) using MSPSO, SVM, and F-score method. Pourpanah et al.^34^ introduced FAM-BSO, a hybrid model for data classification, combining Fuzzy ARTMAP and BSO. Akinola et al.^35^ reviewed metaheuristic algorithms for multiclass feature selection. Agrawal et al.^36^ provided a comprehensive literature analysis on metaheuristic algorithms for feature selection. Peng et al.^37^ proposed Improved TGA (iTGA) for efficient feature selection. Ibrahim et al.^38^ developed SSAPSO, integrating SSA and PSO for feature selection. Abd Elaziz et al.^39^ enhanced Moth-flame Optimization (MFO) with Opposition-based Learning (OBL). Naheed et al.^40^ overviewed medical imaging feature selection approaches. Al-Shourbaji et al.^41^ presented RSA-SO, a concurrent application of Reptile Search Algorithm-Snake Optimizer for feature selection. Mahendru and Agarwal^42^focused on designing a hybrid algorithm for disease diagnosis, exploring various feature selection methods. Xie et al.^43^ introduced two PSO variations to address early convergence and exploitation issues. These metaheuristic algorithms contribute to the ongoing advancements in medical imaging, offering innovative solutions to challenges in optimization and feature selection with implications for disease diagnosis and classification.

### Bone metastasis

Current methods for detecting bone metastases heavily rely on AI-assisted non-invasive diagnostic imaging, addressing the significant complication that affects up to 70% of advanced breast or prostate cancer patients^44^. Various studies explore the application of AI in musculoskeletal radiology, optimizing tasks like exam scheduling and image protocoling^45,46^. Deep learning models for whole-body bone scintigraphy, such as^47^and those by Huang et al.^48^ and Cheng et al.^49^, showcase automated lesion diagnosis and anatomical localization. Radiomics, as discussed by Faiella et al.^50^, proves valuable for determining the stage of bone illness and distinguishing between malignant and benign lesions. Additionally, deep learning methods are evaluated for bone metastasis diagnosis using bone scans^51^. Li et al.^52^ propose a novel framework for identifying scintigraphic images related to lung cancer, emphasizing data preparation and image categorization. Beyond bone scans, Dadgar et al.^53^ compare 18 F-NaF imaging with 99mTc-MDP bone scans and 18 F-fluorodeoxyglucose. Literature analysis by Li et al.^54^ indicates the prevalence of machine learning techniques, with deep learning focusing on bone scintigraphy. Unique approaches, such as X-ray image analysis for cancer stage and grade identification^55^, and evaluating machine learning models for predicting metastases in male breast cancer patients^56^, contribute to the expanding field. Networks for whole-body bone scintigraphy, as proposed by Saito et al.^57^, highlight advancements in evaluation methods. Chen et al.^58^ emphasize the importance of creating a CNN-based diagnostic system for bone metastases segmentation with a limited dataset. In Hodgkin’s lymphoma, Naseri et al.^59^ enhance AI-based techniques for identifying patients with focal skeleton/bone marrow uptake. Li et al.^60^ develop an AI predictive model for colorectal cancer patients prone to bone metastases. Basic web predictors for non-small cell lung cancer bone metastases are explored by Li et al.^61^. Zhou et al.^62^ evaluate machine learning algorithms in classifying bone metastases in lung cancer patients based on demographic factors. Molecular pathways for bone metastases are reviewed by Orcajo-Rincon et al.^63^, focusing on prostate and breast cancer. While existing studies often focus on hot areas requiring segmentation from whole-body scan images, the developed dataset in this investigation supports a robust automated diagnosis model directly applied to whole-body scan images.

## Methods

### The growth optimizer (GO)

GO is a new metaheuristic algorithm for global optimization; The learning and reflection processes individuals use during their social growth activities serve as the main source of inspiration for its design. Learning is the process by which people grow via gaining knowledge from the external world. examining one’s shortcomings and modifying one’s learning techniques to support one’s growth is the process of Reflection^64^.

**(1) Learning phase**:

Four combined gaps are mathematically modeled in the GO learning phase: the gap amidst the leader and the elite ($\:Gap$_1_), the gap amidst the leader and the bottom ($\:Gap$_2_), the gap amidst the elite and the bottom ($\:Gap$_3_), and the gap amidst two random individuals ($\:Gap$_4_).

1 $$\:\left\{\begin{array}{c}{Gap}_{1}={X}_{best}-{X}_{better\:}\\\:{Gap}_{2}={X}_{best}-{X}_{worse}\\\:{Gap}_{3}={X}_{better}-{X}_{worse}\\\:{Gap}_{4}={X}_{L1}-{X}_{L2}\end{array}\right.$$

Equation (1) describes the mathematical model for each collection of gaps. In which $\:{X}_{best}$ stands for the society’s leader, and $\:{X}_{better}\:$represent for one of the next P_1_ − 1 best individuals, denoted as an elite, $\:{X}_{worse}\:$is one of the population’s P_1_ lowest-ranking individuals and is at the bottom of the social hierarchy. Both $\:{X}_{L1}$ and $\:{X}_{L1}$ are different individuals from the ith individuals randomly. The gap among two individuals is measured by$\:\:Gap$_k_ (k = 1, 2, 3, 4).

The learning effect of *LF*_*k*_ on the *k*th group gap will be influenced by it, and *LF*_*k*_ is modeled as follows (Eq. (2)).

The *ith* individual utilizes *SF*_*i*_ to measure his range of acceptable knowledge.

2 $$\:{\:LF}_{K}=\frac{\|{Gap}_{k}\|}{\sum\:_{k=1}^{4}\|{Gap}_{k}\|},\:k=\left(\text{1,2},\text{3,4}\right)$$

where LF_k_ stands for the normalized ratio of the *k*th group gap’s $\:{Gap}_{k}\:$ Euclidean distance, and it has a range of [0,1].

3 $$\:{SF}_{i}=\frac{{GR}_{i}}{{GR}_{max}}$$

In which *GR*_*i*_ is the *i*th individual’s growth resistance and *GR*_*max*_ is the biggest growth resistance of all.

4 $$\:{KA}_{k}=S{F}_{i}\cdot\:L{F}_{k}\cdot\:{Gap}_{k}\:,(k=\text{1,2},\text{3,4})$$

In which $\:{KA}_{k}$is the knowledge gained by the *i*th individual from the gap’s Kth set, $\:S{F}_{i}\:$is an evaluation of its situation, whereas $\:L{F}_{k}\:$is an evaluation of the exterior situation.

Equation (5) provides the ith individual’s specific learning process.

5 $$\:{X}_{i}^{It+1}={X}_{i}^{It}+K{A}_{1}+K{A}_{2}+K{A}_{3}+K{A}_{4}$$

Which *It* is the current iteration’s number an $\:{X}_{i}\:$ is the *i*th individual who grows by assimilating the knowledge acquired throughout the learning phase.

6 $$\:{X}_{i}^{It+1}=\left\{\begin{array}{c}{X}_{i}^{It+1}\:\:\:\:\:\:\:\:\:\:\:\:\:\:\:\:\:\:\:\:if\:f\left({X}_{i}^{It+1}\right)<f\left({X}_{i}^{It}\right)\\\:\left\{\begin{array}{c}{X}_{i}^{It+1}\:\:\:\:\:\:\:\:\:\:\:\:\:\:\:\:\:\:\:\:\:\:\:\:\:if\:{r}_{1}<{p}_{2}\:\:\:\:\:\:\:\:\:\:\:\:\:\:\:\:\:\:\:\:\:\:\:\:\:\:\:\:\:\:\:\:\:\:\:\:\:\\\:{X}_{i}^{It}\:\:\:\:\:\:\:\:\:\:\:\:\:\:\:\:\:\:\:else\:\:\:\:\:\:\:\:\:\:\:\:\:\:\:\:\:\:\:\:\:\:\:\:\:\:\:\:\:\:\:\:\:\:\:\:\:\:\:\end{array}\right.\end{array}\right.$$

The ranking of the *i*th individual in GR ascending order is represented by *i*nd(*i*), where $\:{r}_{1}$ is a uniformly handed out randomized number in the range [0, 1] If the *i*th individual fails to update, P2 decides whether the freshly gained knowledge is retained. $\:{p}_{2}\:$in this instance is 0.001. As a result of the space restriction of Eq. (6), Here is the complete conditional judgment statement for retaining recently obtained knowledge *r*_1_*<* P_2_ & & ind(*i*) ∼= *in*d(1).

This does not simply mean that when an individual update fails, the individual has a 0.001 chance of entering the next-generation population, However, it also guarantees that the current global optimal solution can’t be replaced, or else the algorithm will fail to converge.

**(2) Reflection phase**.

Equations (7) and (8) are used to mathematically describe the reflective process of GO.

7 $$\:{X}_{i,j}^{It+1}=\left\{\begin{array}{c}\left\{\begin{array}{c}Ib+{r}_{4}\times\:\left(ub-Ib\right)\:\:\:if\:{r}_{3}<AF\\\:\:\:\:\:\:\:\:\:\:\:\:\:\:\:\:\:\:\:\:\:\:\:\:\:\:\:\:\:\:\:\:\:\:\:\:\:\:if\:{r}_{2}<{P}_{3}\\\:{X}_{i,j}^{It}+{r}_{5}\times\:\left({R}_{j}-{X}_{i,j}^{It}\right)\:\:\:\:\:\:\:\:else\end{array}\right.\\\:{X}_{i,j}^{It}\:\:\:\:\:\:\:\:\:\:\:\:\:\:\:\:\:\:\:\:\:\:\:\:\:\:\:\:\:\:\:\:\:\:\:\:\:\:\:\:\:\:\:\:\:else\end{array}\right.$$

8 $$\:AF=0.01+0.99\times\:(1-\frac{{FE}_{s}}{Max{FE}_{s}})$$

in which $\:Ib$ and $\:ub$ are the search domain’s upper and lower bounds, and $\:{r}_{2},\:{r}_{3},\:{r}_{4}$, and $\:{r}_{5}\:$are uniformly dispensed randomized numbers in the scope [0,1]. The chance of reflection is determined by the value of $\:{P}_{3}$, which is typically set to 0.3. The maximum number of evaluations (*MaxF*Es) and the current number of evaluations (*F*Es) make up the attenuation factor ($\:AF$).

During the reflection period, the *j*th aspect of the *i*th individual will be guided by some higher-level individual ($\:\text{R}$). The same is true for the learning stage; once the *i*th individual has completed its reflection, it should assess whether it has grown. The entire verification procedure, as well as the following operations, are still being carried out utilizing Eq. (6).

### The arithmetic optimization algorithm

Arithmetic is a fundamental component of number theory and modern mathematics. as well as algebra, analysis, and geometry. Arithmetic operators (i.e., Subtraction, Addition, Division, and Multiplication) are typically the conventional calculation methods used to study numbers; The basic idea for the proposed AOA is to use arithmetic operators to solve arithmetic problems. More details can be found at^65,66^.

### Proposed method

**Fig. 1:**
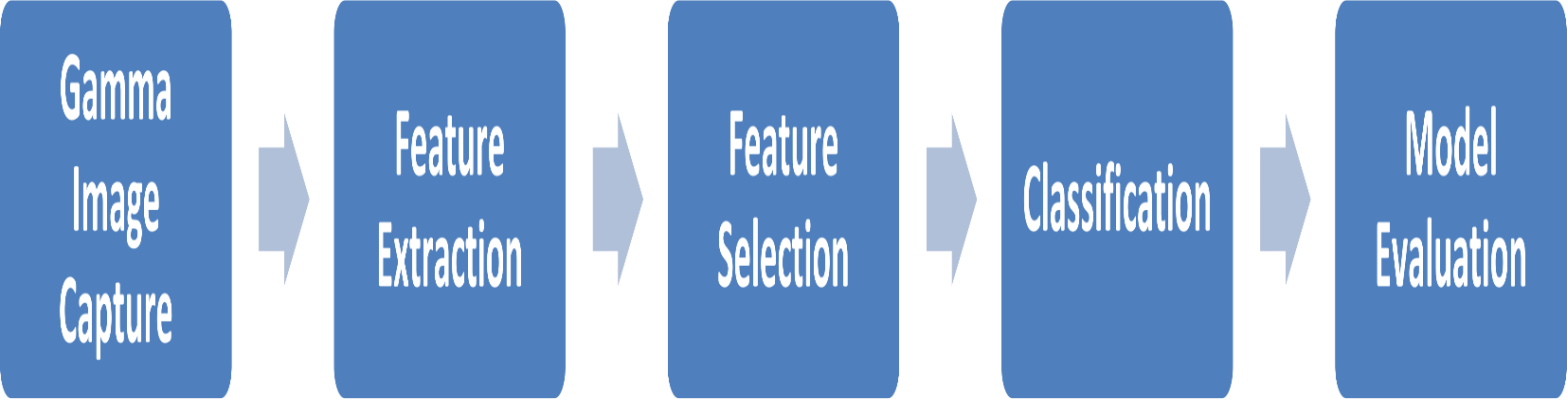
Phases of the proposed method.

There are four phases in the developed method: feature extraction, feature selection, classification, and model evaluation. Segmenting the input image from the gamma-camera scan of the bone was the first step. Then, using machine learning, the features extracted from the segmented image are used to predict the classification of the bone state. The feature extraction and feature selection processes are thoroughly explained in the ensuing subsections. The stages of the suggested method are depicted in Fig. 1.

#### **Feature extraction phase**

Given the high demand for deploying deep learning models on the edge and mobile devices, the creation of models that fit in mobile platforms presents notable challenges in terms of model size and processing speed, mainly when dealing with medical image processing applications. To overcome these challenges, we integrate a Mobile Vision Transformer (MobileViT) model^67,68^ in our framework to capture highly complex representations from raw medical imagery using two primary components including ViT and lightweight CNN featuring a limited number of parameters.

**Fig. 2:**
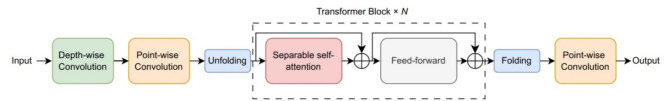
The MobileViTv2 block architecture components.

In our framework, we harnessed the power of MobileViTv2 as the fundamental feature extraction module. MobileViTv2 introduces a separable self-attention mechanism with a linear complexity approach, while the traditional MobileViT model relies on the (MHA) multi-headed self-attention mechanism. Thus, MobileViTv2 can diminish the computational complexity and possess a fast latency with fewer training parameters. The model is a core component in both the training process and the extraction of image feature representations. The input images were resized to a uniform resolution of 244 × 244 pixels for the training and feature extraction, as depicted in Fig. 2.

**(1) Depth-wise Separable Convolution (DSC)**.

This architectural novelty in MobileViTv2 enables it to perform computations based on the number of image patches using element-wise operations, as opposed to matrix multiplications employed in traditional self-attention mechanisms. In contrast to the conventional convolutional approach that contains three phases (unfolding, local processing (matrix multiplication), and folding) for capturing global features, the MobileViTv2 block leverages transformer-based global processing instead of local processing through convolutions (unfolding, transformers stack, and folding). In addition, the DSC block possesses two convolution operations, including depth-wise convolution and point-wise convolution. This synergy of CNN and ViT properties empowers the MobileViT block to learn more efficient image representations while employing fewer trainable parameters.

**(2) Separable Self-Attention Mechanism**.

Compared to traditional self-attention mechanisms, which possess a quadratic complexity related to input sequence length, MobileViTv2 incorporates a separable self-attention mechanism to overcome this issue. The MobileViTv2 incorporates self-attention separately for each head, which converts the complexity to linear using element-wise operations to compute attention instead of traditional matrix multiplications. Equation 14 defines the mathematical representation of self-attention where V, *K*, and Q, represent value matrices, key, and query respectively. The key vectors’ dimensions are represented by the *dk*

9 $$\:Attention\left(Q,\:K,\:V\right)=Softmax\left(\frac{Q\left({K}^{T}\right)}{\sqrt{{d}_{k}}}\right)\cdot V$$

**(3) MobileViTv2 Block**.

The MobileViTv2 core building blocks comprise a separable self-attention mechanism, residual connections, and feed-forward networks. In addition, the DSC block captures both local and global information in the data. For instance, MobileViTv2 introduces the inverted residuals, which reverse the traditional structure to narrow-to-wide-to-narrow instead of wide-to-narrow-to-wide structure by incorporating the depth-wise convolution operations. Meanwhile, linear bottlenecks are employed to produce a linear output to overcome the challenges in the inverted residual structure. The MobileViTv2 stacks several transformer blocks to process the flattened patches as visual tokens and model global dependencies.

Before the last classification layer, we perform feature extraction of the learned representations for each image as a feature vector of size 1 × 256, which will be fed to the proposed feature selection algorithm in our framework for further processing. During the training process, The MobileViTv2 model was fine-tuned for 100 epochs with the early stopping mechanism on an NVIDIA GTX1080 GPU. A batch of size 16 and 0.001 learning rate were selected as the network training hyper-parameters where AdamW optimizer was used.

#### Proposed feature selection algorithm

**Fig. 3:**
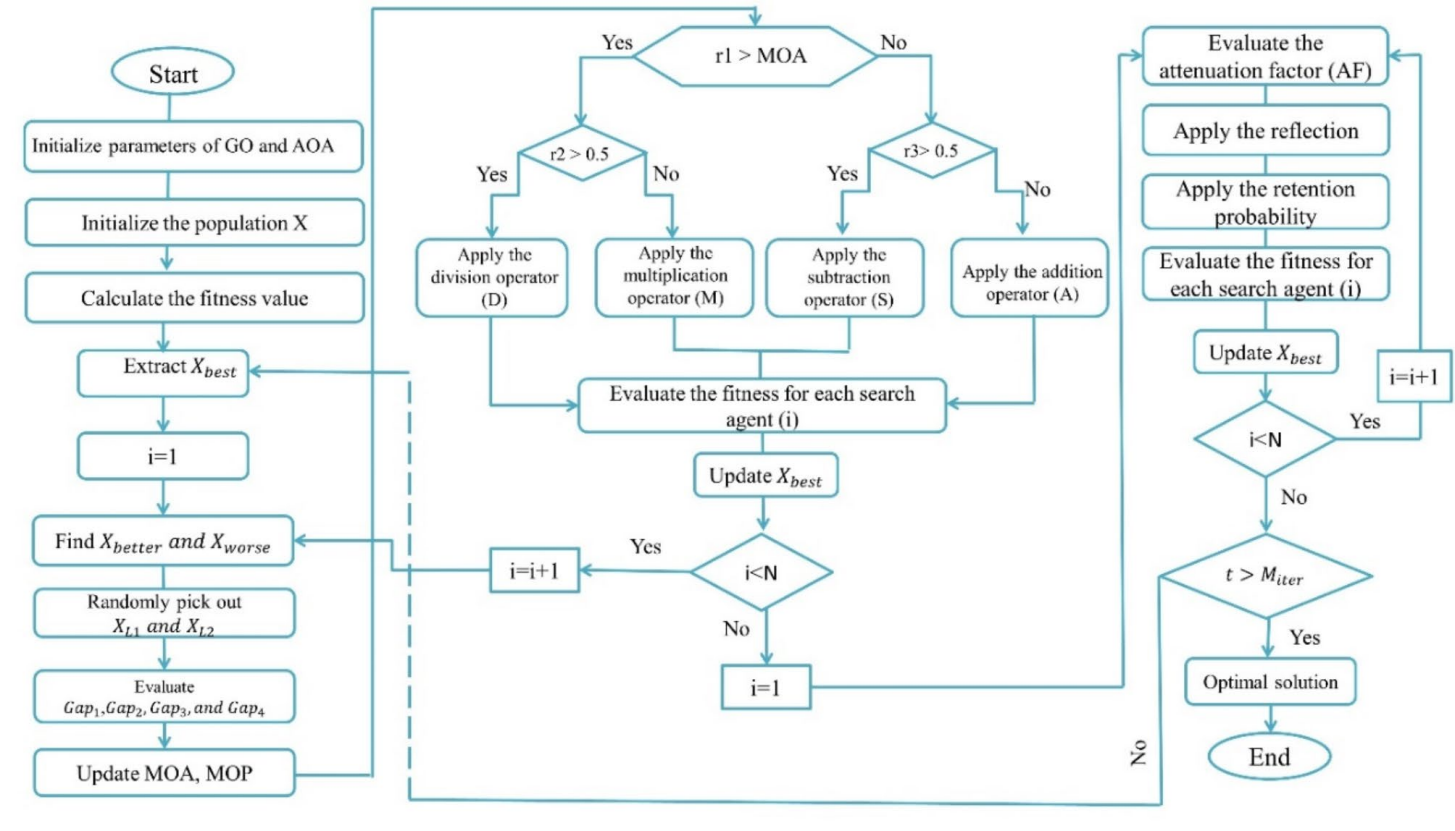
The proposed algorithm’s main structure.

The main phases of the suggested FS method are presented in this section. as shown in Fig. 3. The goal of the GO-AOA approach presented here is to improve GO’s exploring ability by utilizing the AOA method.

To start, the GO-AOA divides the input dataset into training and testing sets, which correspond to 80% and 20% of the input data instances, respectively. Next, for the provided problem (feature selection), define the parameters and create a population that represents a set of individuals. Then initialize the population $\:X$ by using the following formula:

10 $$\:X=lb+\left(ub-lb\right)\times\:rand\left(N,D\right),\:i=1,\dots\:.,N$$

where *N* shows population size. *D* stands for the population dimension. $\:ub$ and $\:lb$ refer to the upper and lower and bound.

In the second stage (the loop iteration process), to obtain an ascending ranking array of the complete population, first sort the individuals by growth resistance (*GR*). This information is then stored in *ind*. Then the best fitness value ($\:{X}_{best}$) is assigned, the current iteration’s optimal solution ($\:{X}_{best}$) is updated before each iteration, and the real-time global optimal solution $\:\left(gbestx\right)$ is updated at each evaluation. In general, the fitness value is computed using the following formula:

11 $$\:Fi{t}_{i}=\alpha\:{E}_{i}+\left(1-\alpha\:\right)\frac{\left|B{X}_{i}\right|}{D}$$

12 $$\:B{X}_{i}=\left\{\begin{array}{c}1\:\:\:\:\:\:\:if\:{X}_{i}\ge\:0.5\:\\\:0\:\:\:\:\:otherwise\:\end{array}\:\:\:\:\right.$$

where $\:\alpha\:\in\:\left[\text{0,1}\right]$ refers to random value balance between the error of classification $\:{E}_{i}$ and the ratio of selected features (i.e., $\:\frac{\left|B{X}_{i}\right|}{D}$). The value of $\:\left|B{X}_{i}\right|$ refers to the number of selected features which corresponding to ones in the Boolean vector $\:B{X}_{i}$ of $\:{X}_{i}$ that defined in Eq. (12).

**Exploration phase**: first the $X_{better}$ and one $X_{worse}$ will be selected to participate and compute the $GAP_{k}, \, LF_{k}, \, SF_{i}, {\text{and}}\, KA_{k}$ in Eqs. (1)–(4) then switching the exploration phase of the GO with the exploration phase of AOA, This process is formulated using Eqs. (10)–(12).

**Exploitation phase**: Equations (7) and (8) will be utilized by the algorithm to process the *j*th dimension for the *i*th individual; the *i*th individual and the real-time global optimal solution (*gbestx*) are then updated using Eq. (6).

In Eq. (8) we change the value of AF to be:

13 $$\:AF=0.02+0.33\times\:(1-\frac{It}{M\_Iter})$$

**Termination criterion**: If the current number of evaluations (*It*) attains the maximum number of iterations ($M_{iter}$) then return the *gbestx* and stop the program otherwise turn to the loop iteration process. The pseudo-code for the proposed model is given in Algorithm 1.

**Figure:**
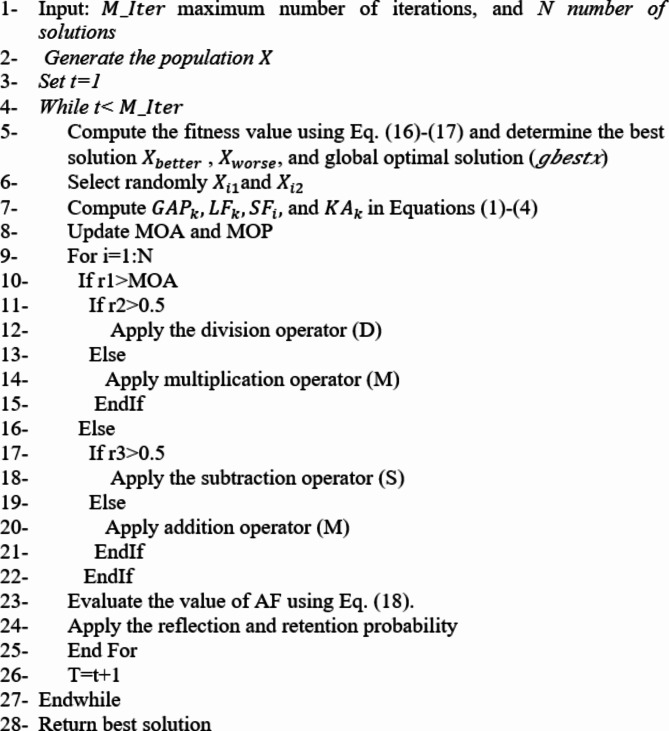
**Algorithm 1** Step of proposed algorithm.

### The complexity of the GOAOA

Within this part, we discuss the complexity of the developed GOAOA model as feature selection method. The complexity of GOAOA depends on the number of solutions $\:N$, number of features $\:D$, and the total number of iterations $\:M\_Iter$.

$$\:O\left(GOAOA\right)=N\times\:D+\left(N\times\:D+N\times\:D+N\times\:Coff+N\times\:D\right)\times\:M\_Iter\:\:$$

$$\:O\left(GOAOA\right)=N\times\:D+\left(3 N\times\:D+N\times\:Coff\right)\times\:M\_Iter$$

where $\:Coff$ refers to the number of fitness evaluation.

## Experimental results and discussion

### Parameter settings and performance metrics

This section evaluates the suggested GOAOA approach for choosing significant features using twenty datasets. The GOAOA findings are compared to six approaches: Whale Optimization Algorithm (WOA)^69^, Harris Hawks optimization (HHO)^25^, Salp swarm algorithm (SSA)^30^, Binary Particle Swarm Optimization (BPSO)^70^, Growth Optimizer GO^64^, and The Arithmetic Optimization Algorithm (AOA)^65^. These methods’ parameter settings are determined based on their representations in the original work, and they are presented in Table 1.

**Table 1:** The value of each algorithm’s parameter.

Algorithm	Parameter	Value
BPSO	W, c1, c2,	W = 2, C1 = 2, C2 = 2,
WOA	b	1
SSA	C1, C2	C1 € [01], C2 € [01]
GO	P1, P2, P3	5, 0.001, 0.3
AOA	Alpha, µ	5, 0.5
GOAOA	Alpha, µ, P1, P2, P3	5, 0.5, 10, 0.004, 0.3
HHO	E, beta	E$\:\in\:$ [0.2], beta = 1.5

Furthermore, seven performance metrics are used: the standard deviation (Std), average (Avg), maximum (Max), and minimum (Min) of the objective function’s value. In addition, the specificity, sensitivity, and accuracy are calculated as introduced in Eqs. (14) and (18), respectively.

14 $$\:\text{A}\text{c}\text{c}\text{u}\text{r}\text{a}\text{c}\text{y}\:=\:\frac{TP+TN}{TP+TN+FP+FN}$$

15 $$\:\text{S}\text{e}\text{n}\text{s}\text{i}\text{t}\text{i}\text{v}\text{i}\text{t}\text{y}\:=\:\frac{TP}{TP+FN}$$

16 $$\:\text{S}\text{p}\text{e}\text{c}\text{i}\text{f}\text{i}\text{c}\text{i}\text{t}\text{y}\:=\:\frac{TN}{TN+FP}$$

where TP = true positive, TN = true negative, FP = false positive, and FN = false negative.

17 $$\:\text{S}\text{t}\text{a}\text{n}\text{d}\text{a}\text{r}\text{d}\:\text{D}\text{e}\text{v}\text{i}\text{a}\text{t}\text{i}\text{o}\text{n}\:=\:\sqrt{\frac{1}{N-1}\sum\:_{i=1}^{N}{({X}_{i}-\mu\:)}^{2}}$$

$\:N$ is the number of the observations, $\:{X}_{i}$ is *i*th observation, $\:\mu\:$ is a mean of observation.

Average (Avg): The average can be identified by summing up all the numbers and then dividing them by the number of observations.

18 $$\:Average\:=\frac{1}{N}\sum\:_{i=1}^{N}{X}_{i}$$

### Results and discussion using UCI datasets

The UCI dataset (https://tinyurl.com/UCIDS) is a data repository maintained and made available by the University of California, the dataset covers a wide range of fields and topics, including but not limited to medicine, biology, social sciences, physics, engineering, among others, this repository encapsulates a rich array of data derived from various domains and sources.

In our investigation, we employed thirteen UCI datasets to evaluate the performance of the GOAOA algorithm and compared it with alternative algorithms. Table 2 presents the average objective function values across these datasets. The BPSO algorithm emerged as the top performer, securing the first position in Table 2 by exhibiting superior performance in four out of the thirteen datasets specifically, Breastcancer4, Vote3, WineEW1, and zoo. Following closely, the GOAOA algorithm secured the second position, demonstrating its efficacy by achieving the best average performance in three datasets: BreastEW, SonarEW2, and WaveformEW9. The WOA claimed the third rank, outperforming others in two datasets, namely Brain_T21 and leuk1. The CS secured the fourth position, excelling in the PenglungEW3 dataset.

**Table 2:** Average of the fitness function values.

Dataset name	BPSO	WOA	HHO	SSA	GO	AOA	GOAOA
base_Brain_T21	0.11173	**0.01061**	0.04947	0.19631	0.35058	0.03372	0.04835
base_Breastcancer4	**0.05141**	0.05904	0.05298	0.05498	0.07794	0.05194	0.05561
base_BreastEW	0.05217	0.05653	0.05474	0.06692	0.11429	0.04987	**0.04578**
base_CongressEW1	**0.0373**	**0.0373**	0.04104	0.0706	0.12896	0.04603	**0.0373**
base_Exactly	**0.0462**	0.10369	0.06071	0.08157	0.14139	**0.0462**	0.0716
base_leuk1	0.07823	**0.00728**	0.01037	0.11806	0.13583	0.01669	0.02087
base_M-of-n3	**0.0462**	0.08614	0.05000	0.06061	0.13283	**0.0462**	0.05546
base_PenglungEW3	0.037111	0.00988	**0.00802**	0.05182	0.07441	0.01445	0.01464
base_SonarEW2	0.04748	0.06598	0.05619	0.07412	0.11871	0.05107	**0.03557**
base_Vote3	**0.01439**	0.02851	0.02337	0.04303	0.07465	0.02063	0.01801
base_WaveformEW9	0.26018	0.28587	0.27475	0.28984	0.31842	0.26181	**0.24994**
base_WineEW1	**0.02926**	0.06098	0.03253	0.05004	0.08921	0.03964	0.04272
zoo	**0.03309**	0.04251	0.04001	0.04566	0.06315	0.03372	0.03624

Boldface indicates the best value.

Table 3 displays the standard deviation results for the algorithms. From this table we can see that BPSO had the lowest values across the majority of datasets (7 out of 13), earning it the first rank. AOA came in second, showing favorable stability in two datasets, while the proposed GOAOA method yielded competitive standard deviation results compared to other methods.

**Table 3:** STD results for fitness value.

Dataset name	BPSO	WOA	HHO	SSA	GO	AOA	GOAOA
base_Brain_T21	0.043059	0.028111	0.045693	0.045381	0.072929	**0.026614**	0.04347
base_Breastcancer4	**0.000821**	0.004306	0.001386	0.00612	0.008138	0.001471	0.004225
base_BreastEW	**0.003138**	0.011463	0.006794	0.006618	0.006045	0.02374	0.006465
base_CongressEW1	**0.00000**	**0.00000**	0.005265	0.006641	0.012799	0.027607	**0.00000**
base_Exactly	**7.31E-18**	0.086511	0.030077	0.051884	0.069916	**7.31E-18**	0.080322
base_leuk1	0.031171	0.018812	0.010917	**0.000217**	0.00653	0.002965	0.000374
base_M-of-n3	**7.31E-18**	0.042123	0.004006	0.015582	0.026639	**7.31E-18**	0.029283
base_PenglungEW3	**0.00079**	0.009877	0.004449	0.001124	0.003295	0.002227	0.001778
base_SonarEW2	**0.006713**	0.020423	0.0107	0.017035	0.025487	0.027681	0.008913
base_Vote3	**0.003043**	0.014666	0.008141	0.012394	0.011084	0.019554	0.006515
base_WaveformEW9	0.008732	0.015558	**0.006404**	0.00704	0.012358	0.025167	0.012426
base_WineEW1	**0.006074**	0.026728	0.010594	0.018001	0.020561	0.020956	0.011848
zoo	**0.003043**	0.007117	0.006068	0.006631	0.011934	0.004405	0.00497

Boldface indicates the best value.

Additionally, Table 4 reveals the worst fitness function values, with BPSO leading in most datasets, securing the top rank. Following closely, GOAOA claimed the second position, showcasing competitive performance. The WOA method secured the third rank whereas, the AOA came in the fourth rank.

**Table 4:** Maximum fitness value results.

Dataset name	BPSO	WOA	HHO	SSA	GO	AOA	GOAOA
base_Brain_T21	0.1388	**0.0905**	0.0962	0.2416	0.4401	0.108	0.1115
base_Breastcancer4	**0.0526**	0.0655	0.0544	0.0702	0.0924	0.0544	0.0625
base_BreastEW	**0.0526**	0.0655	0.0544	0.0702	0.0924	0.0544	0.0625
base_CongressEW1	**0.0373**	**0.0373**	0.0498	0.081	0.1453	0.1246	**0.0373**
base_Exactly	**0.0462**	0.2691	0.1457	0.2151	0.2633	**0.0462**	0.3002
base_leuk1	**0.0462**	0.2691	0.1457	0.2151	0.2633	**0.0462**	0.3002
base_M-of-n3	**0.0462**	0.2691	0.1457	0.2151	0.2633	**0.0462**	0.3002
base_PenglungEW3	0.0382	0.0372	**0.0178**	0.0532	0.0791	0.0191	**0.0178**
base_SonarEW2	0.0631	0.0943	0.0681	0.0979	0.1493	0.1262	**0.0531**
base_Vote3	**0.0188**	0.0563	0.0338	0.0563	0.0963	0.075	0.0275
base_WaveformEW9	**0.2707**	0.3071	0.2808	0.3025	0.3296	0.3296	0.2736
base_WineEW1	**0.0385**	0.1019	0.0558	0.0712	0.1096	0.0942	0.0635
zoo	**0.0375**	0.0500	0.0500	0.0563	0.075	0.0438	0.0438

The best values are highlighted in bold.

Table 5 presents the best fitness function results for all algorithms. The GOAOA exhibited the lowest values compared to other algorithms, consistently achieving superior performance across the majority of cases. The HHO demonstrated the second-best performance, followed by the WOA in the third position, and subsequently, BPSO, AOA, and SSA.

**Table 5:** Minimum fitness value results.

Dataset name	BPSO	WOA	HHO	SSA	GO	AOA	GOAOA
base_Brain_T21	0.0489	0.0002	**0**	0.1508	0.2538	0.0188	0.0201
base_Breastcancer4	**0.0509**	0.0544	**0.0509**	**0.0509**	0.0637	0.0509	**0.0509**
base_BreastEW	0.0479	0.0425	0.0404	0.057	0.1061	0.0358	**0.0312**
base_CongressEW1	**0.0373**	**0.0373**	**0.0373**	0.0623	0.1039	**0.0373**	**0.0373**
base_Exactly	**0.0462**	**0.0462**	**0.0462**	**0.0462**	0.0705	**0.0462**	**0.0462**
base_leuk1	0.0483	**0.0001**	**0.0001**	0.1176	0.1182	0.0093	0.0201
base_M-of-n3	**0.0462**	**0.0462**	**0.0462**	0.0538	0.0917	**0.0462**	**0.0462**
base_PenglungEW3	0.0357	**0.0043**	**0.0034**	0.0502	0.0689	0.0117	0.0117
base_SonarEW2	0.04	0.0267	0.035	0.045	0.0748	0.0283	**0.0233**
base_Vote3	**0.0125**	**0.0125**	**0.0125**	0.0188	0.0588	**0.0125**	**0.0125**
base_WaveformEW9	0.2452	0.2603	0.2634	0.2789	0.2895	0.2417	**0.2259**
base_WineEW1	**0.0231**	**0.0231**	**0.0231**	**0.0231**	0.0385	**0.0231**	**0.0231**
zoo	**0.0312**	**0.0312**	**0.0312**	0.0375	0.0438	**0.0312**	**0.0312**

The best values are highlighted in bold.

**Fig. 4:**
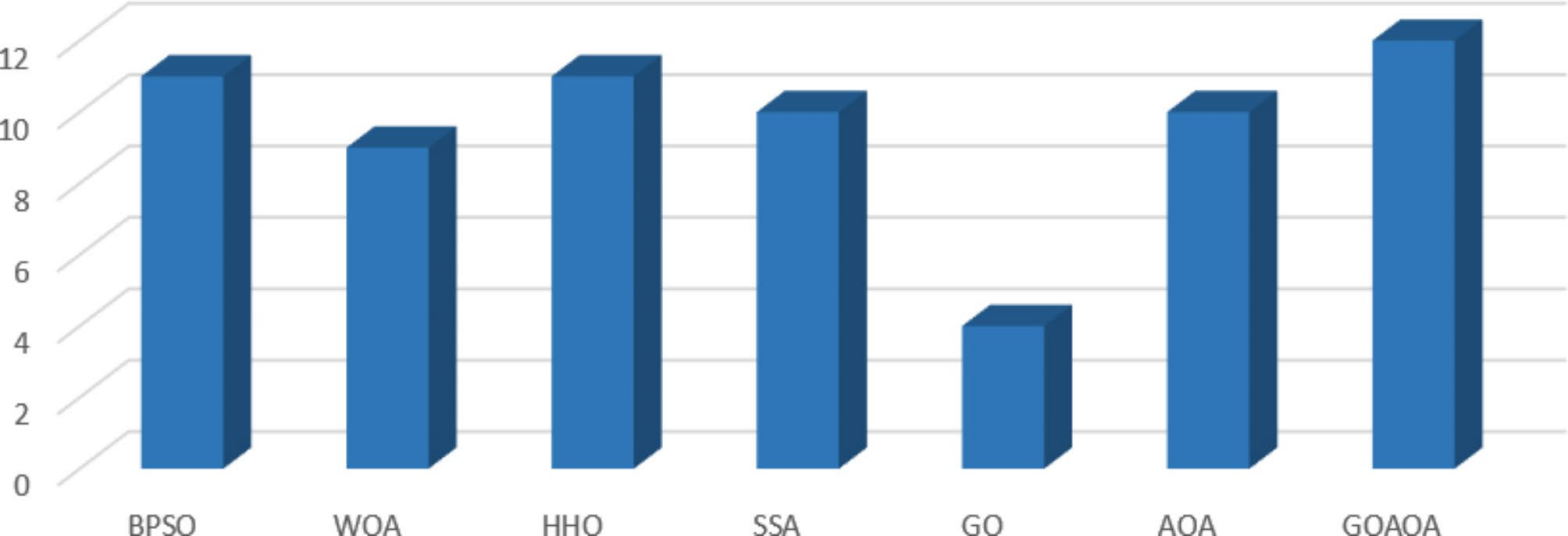
Count of datasets where each algorithm obtained the highest values.

Furthermore, we employed three classification measures to assess the efficacy of the proposed algorithm. The accuracy measure findings in Table 6 highlight the consistent superior performance of the developed GOAOA across all datasets. The HHO and BPSO secured the second position, while AOA and SSA followed closely in third place, each achieving the highest score in 10 out of 13 datasets. WOA occupied the fourth position. It is noteworthy that the GO techniques demonstrated the lowest classification accuracy values. Figure 4 shows the count of datasets where each algorithm obtained the highest values.

**Table 6:** Results of accuracy for FS technique.

Dataset name	BPSO	WOA	HHO	SSA	GO	AOA	GOAOA
base_Brain_T21	**1.000**	**1.000**	**1.000**	0.900	0.800	**1.000**	**1.000**
base_Breastcancer4	**0.993**	0.971	**0.993**	**0.993**	0.986	**0.993**	**0.993**
base_BreastEW	**0.991**	0.983	0.983	**0.991**	0.965	0.983	**0.991**
base_CongressEW1	0.966	0.966	0.966	**0.977**	0.954	0.966	0.966
base_Exactly	**1.000**	**1.000**	**1.000**	**1.000**	0.990	**1.000**	**1.000**
base_leuk1	**1.000**	**1.000**	**1.000**	0.933	0.933	**1.000**	**1.000**
base_M-of-n3	**1.000**	**1.000**	**1.000**	**1.000**	0.975	**1.000**	**1.000**
base_PenglungEW3	**1.000**	**1.000**	**1.000**	**1.000**	**1.000**	**1.000**	**1.000**
base_SonarEW2	**1.000**	**1.000**	**1.000**	**1.000**	0.976	**1.000**	**1.000**
base_Vote3	**1.000**	**1.000**	**1.000**	**1.000**	**1.000**	**1.000**	**1.000**
base_WaveformEW9	0.772	0.747	**0.774**	0.758	0.740	0.770	**0.774**
base_WineEW1	**1.000**	**1.000**	**1.000**	**1.000**	**1.000**	**1.000**	**1.000**
zoo	**1.000**	**1.000**	**1.000**	**1.000**	**1.000**	**1.000**	**1.000**

Bold indicates the best value.

Table 7 displays the results of the sensitivity measure. Remarkably, both GOAOA and SSA claimed the top position, achieving the highest sensitivity in 12 out of 13 datasets. Following closely, AOA, BPSO, and HHO secured the second position, with nearly equal results, each attaining the highest sensitivity in 11 out of 13 datasets. WOA and GO occupied the third and fourth positions, respectively.

**Table 7:** Results of each FS method’s sensitivity.

Dataset name	BPSO	WOA	HHO	SSA	GO	AOA	GOAOA
base_Brain_T21	**1.000**	**1.000**	**1.000**	1.000	1.000	**1.000**	**1.000**
base_Breastcancer4	**1.000**	0.989	**1.000**	**1.000**	0.989	**1.000**	**1.000**
base_BreastEW	**1.000**	1.000	1.000	**1.000**	1.000	1.000	**1.000**
base_CongressEW1	0.963	0.963	0.963	**0.982**	0.944	0.963	0.963
base_Exactly	**1.000**	**1.000**	**1.000**	**1.000**	1.000	**1.000**	**1.000**
base_leuk1	**1.000**	**1.000**	**1.000**	1.000	1.000	**1.000**	**1.000**
base_M-of-n3	**1.000**	**1.000**	**1.000**	**1.000**	0.970	**1.000**	**1.000**
base_PenglungEW3	**1.000**	**1.000**	**1.000**	**1.000**	**1.000**	**1.000**	**1.000**
base_SonarEW2	**1.000**	**1.000**	**1.000**	**1.000**	0.952	**1.000**	**1.000**
base_Vote3	**1.000**	**1.000**	**1.000**	**1.000**	**1.000**	**1.000**	**1.000**
base_WaveformEW9	0.772	0.722	**0.778**	0.772	0.746	0.775	**0.781**
base_WineEW1	**1.000**	**1.000**	**1.000**	**1.000**	**1.000**	**1.000**	**1.000**
zoo	**1.000**	**1.000**	**1.000**	**1.000**	**1.000**	**1.000**	**1.000**

Bold indicates the best value.

Table 8 illustrates that BPSO and HHO yielded the highest results in the specificity metric, securing the top rank with the highest scores in 11 out of 13 datasets. Following closely, GOAOA, AOA, and WOA obtained the second position, while GO claimed the third. These outcomes suggest that the proposed GOAOA exhibits the capability to effectively categorize diverse dataset types, achieving competitive results in specificity across the evaluated datasets.

**Table 8:** Results of each FS method’s specificity.

Dataset name	BPSO	WOA	HHO	SSA	GO	AOA	GOAOA
base_Brain_T21	**1.000**	**1.000**	**1.000**	**1.000**	0.750	**1.000**	**1.000**
base_Breastcancer4	0.980	0.940	0.980	**1.000**	0.980	0.980	0.980
base_BreastEW	**1.000**	0.976	**1.000**	0.976	0.951	0.976	0.976
base_CongressEW1	**0.970**	**0.970**	**0.970**	**0.970**	**0.970**	**0.970**	**0.970**
base_Exactly	**1.000**	**1.000**	**1.000**	**1.000**	0.969	**1.000**	**1.000**
base_leuk1	**1.000**	**1.000**	**1.000**	0.900	0.900	**1.000**	**1.000**
base_M-of-n3	**1.000**	**1.000**	**1.000**	**1.000**	0.985	**1.000**	**1.000**
base_PenglungEW3	**1.000**	**1.000**	**1.000**	**1.000**	**1.000**	**1.000**	**1.000**
base_SonarEW2	**1.000**	**1.000**	**1.000**	**1.000**	**1.000**	**1.000**	**1.000**
base_Vote3	**1.000**	**1.000**	**1.000**	**1.000**	**1.000**	**1.000**	**1.000**
base_WineEW1	**1.000**	**1.000**	**1.000**	**1.000**	**1.000**	**1.000**	**1.000**
zoo	**1.000**	**1.000**	**1.000**	**1.000**	**1.000**	**1.000**	**1.000**

Bold indicates the best value.

The statistical outcomes of each feature selection technique, as determined by the Friedman test, are presented in Table 9. Notably, the GOAOA algorithm secured the highest rank in both accuracy and sensitivity measures. In the specificity measure, HHO and BPSO achieved the highest rank, with GOAOA and AOA following closely in the second position.

**Table 9:** Friedman test results of each FS method.

Mean rank	BPSO	WOA	HHO	SSA	GO	AOA	GOAOA
Accuracy	4.5769	3.8077	4.4615	4.1538	2.0385	4.2692	**4.6923**
Sensitivity	4.1154	3.6538	4.3077	4.3462	2.9615	4.2308	**4.3846**
Specificity	**4.4167**	3.9167	**4.4167**	4.125	2.7917	4.1667	4.1667

The best values are highlighted in bold.

### Parameter analysis of GOAOA

**Fig. 5:**
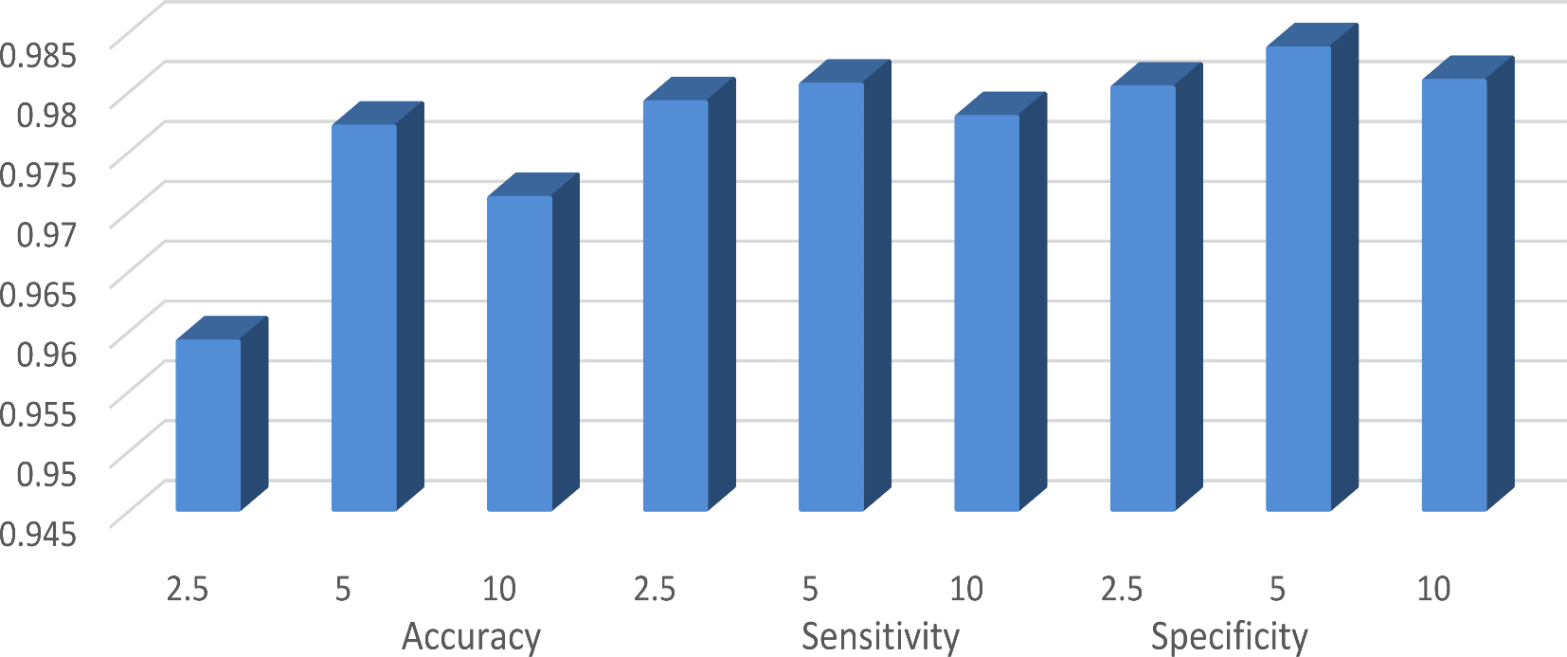
Average performance for various values of parameter p1.

In this section, we explore the influence of varying the parameters of GOAOA (p1 and α) on its performance. Tables 10 and 11 present the results of this experimental analysis, where we examine the impact of different values for p1 (2, 5, and 10) and α values of (2.5, 5, and 10).

that the optimal accuracy is achieved when the value of p1 is set to 5. Interestingly, this high accuracy is consistent across approximately 5 datasets for all p1 values. Moreover, for sensitivity, the best performance is observed at p1 = 5, with minimal variations in results for different p1 values. On the other hand, specificity yields the best outcome when p1 is set to 10. Figure 5 depicts the average performance across the evaluated datasets.

**Table 10:** The outcomes of various p1 parameter values.

	Accuracy			Sensitivity			Specificity		
	2	5	10	2	5	10	2	5	10
base_Brain_T21	0.800	**1.000**	**1.000**	**1.000**	**1.000**	**1.000**	**1.000**	**1.000**	**1.000**
base_Breastcancer4	**0.979**	**0.979**	0.971	0.990	**1.000**	**1.000**	0.947	0.949	**0.959**
base_BreastEW	0.974	**0.983**	**0.983**	0.987	**1.000**	**1.000**	0.975	0.977	**0.978**
base_CongressEW1	**0.989**	**0.989**	0.954	**1.000**	0.979	0.952	0.971	**1.000**	0.960
base_Exactly	**1.000**	**1.000**	**1.000**	**1.000**	**1.000**	**1.000**	**1.000**	**1.000**	**1.000**
base_leuk1	**1.000**	**1.000**	**1.000**	**1.000**	**1.000**	**1.000**	**1.000**	**1.000**	**1.000**
base_M-of-n3	**1.000**	**1.000**	**1.000**	**1.000**	**1.000**	**1.000**	**1.000**	**1.000**	**1.000**
base_PenglungEW3	**1.000**	**1.000**	**1.000**	**1.000**	**1.000**	**1.000**	**1.000**	**1.000**	**1.000**
base_SonarEW2	**1.000**	0.976	**1.000**	**1.000**	**1.000**	**1.000**	**1.000**	**1.000**	**1.000**
base_Vote3	0.967	**1.000**	0.983	**1.000**	**1.000**	**1.000**	0.975	**1.000**	0.974
base_WaveformE9	0.761	**0.776**	0.761	0.752	**0.769**	0.761	0.876	0.862	**0.880**
base_WineEW1	**1.000**	**1.000**	0.972	**1.000**	**1.000**	**1.000**	**1.000**	**1.000**	**1.000**
zoo	**1.000**	**1.000**	**1.000**	**1.000**	**1.000**	**1.000**	**1.000**	**1.000**	**1.000**

The best values are highlighted in bold.

Table 11 also includes the results of GOAOA with varied values for parameter α (2.5, 5, and 10). The following observations are made. To begin, an α value of 5 results in the optimal overall performance. Moreover, altering the value of alpha has no substantial effect on the outcome.

**Fig. 6:**
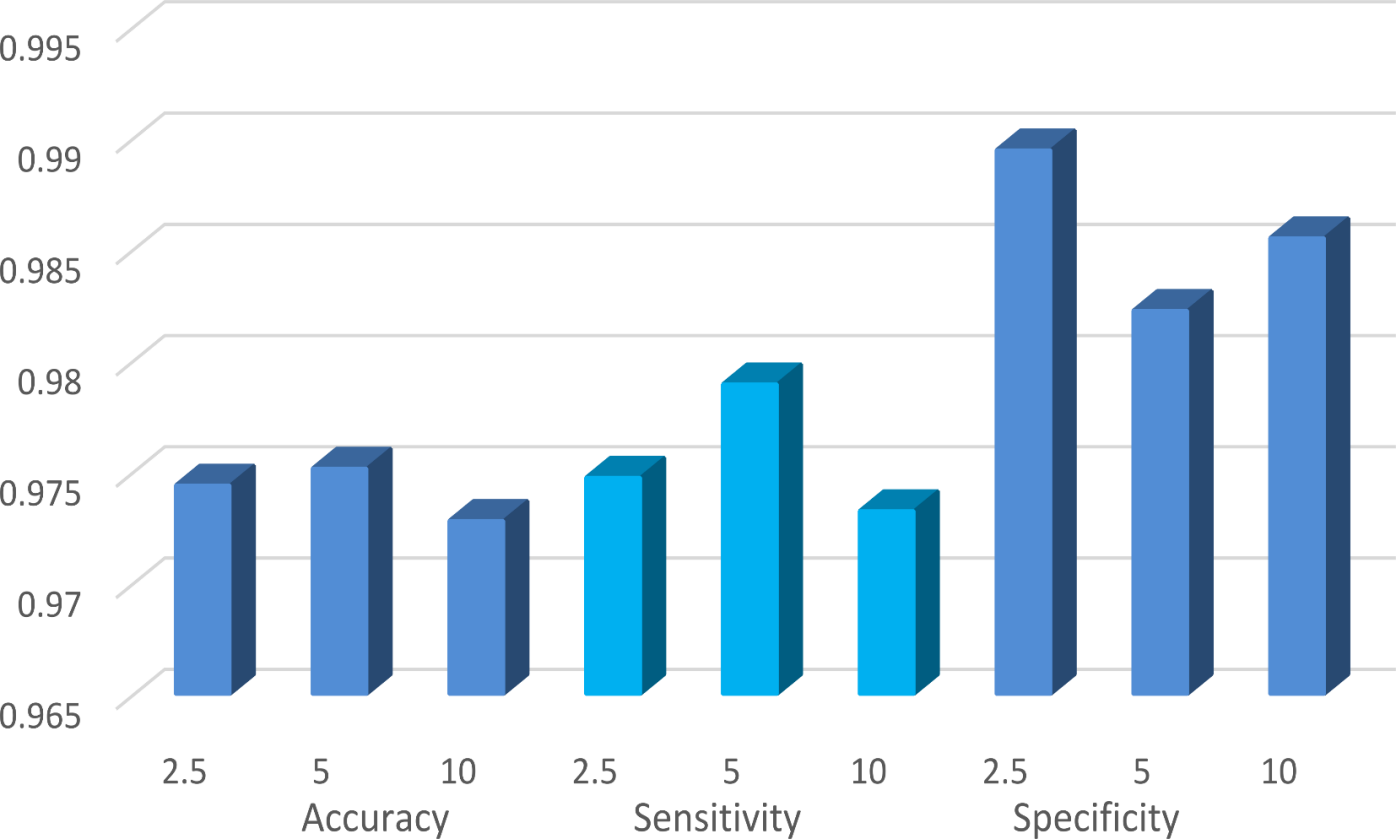
Average performance at various values of the parameter Alpha.

Figure 6 displays the average performance across the investigated datasets when the value of α is altered. Importantly, changing one parameter while keeping the other constant has no discernible effect on the outcome.

**Table 11:** The outcomes of various alpha parameter values.

Alpha	Accuracy			Sensitivity			Specificity		
	2.5	5	10	2.5	5	10	2.5	5	10
base_Brain_T21	**1.000**	1.000	1.000	1.000	1.000	1.000	1.000	1.000	1.000
base_Breastcancer4	0.971	0.979	**0.971**	**0.980**	1.000	0.980	**1.000**	**0.949**	0.974
base_BreastEW	**1.000**	0.983	0.983	**1.000**	1.000	1.000	**1.000**	**0.977**	1.000
base_CongressEW1	0.977	0.989	**0.989**	0.979	**0.979**	**1.000**	**1.000**	1.000	**0.970**
base_Exactly	1.000	1.000	1.000	1.000	1.000	1.000	1.000	1.000	1.000
base_leuk1	1.000	1.000	1.000	1.000	1.000	1.000	1.000	1.000	1.000
base_M-of-n3	1.000	1.000	1.000	1.000	1.000	1.000	1.000	1.000	1.000
base_PenglungEW3	1.000	1.000	1.000	1.000	1.000	1.000	1.000	1.000	1.000
base_SonarEW2	1.000	**0.976**	0.976	1.000	1.000	0.958	1.000	1.000	1.000
base_Vote3	**0.983**	1.000	**1.000**	1.000	1.000	1.000	**1.000**	1.000	**1.000**
base_WaveformEW9	**0.761**	0.776	**0.755**	**0.739**	0.769	**0.740**	**0.874**	**0.862**	0.882
base_WineEW1	1.000	1.000	**1.000**	1.000	1.000	1.000	1.000	1.000	1.000
zoo	1.000	1.000	1.000	1.000	1.000	1.000	1.000	1.000	0.933

The best values are highlighted in bold.

### Applicability of the proposed model to predict bone metastasis

The effectiveness of GOAOA was further explored using real medical data, as outlined in “Data description”.

#### Data description

**Fig. 7:**
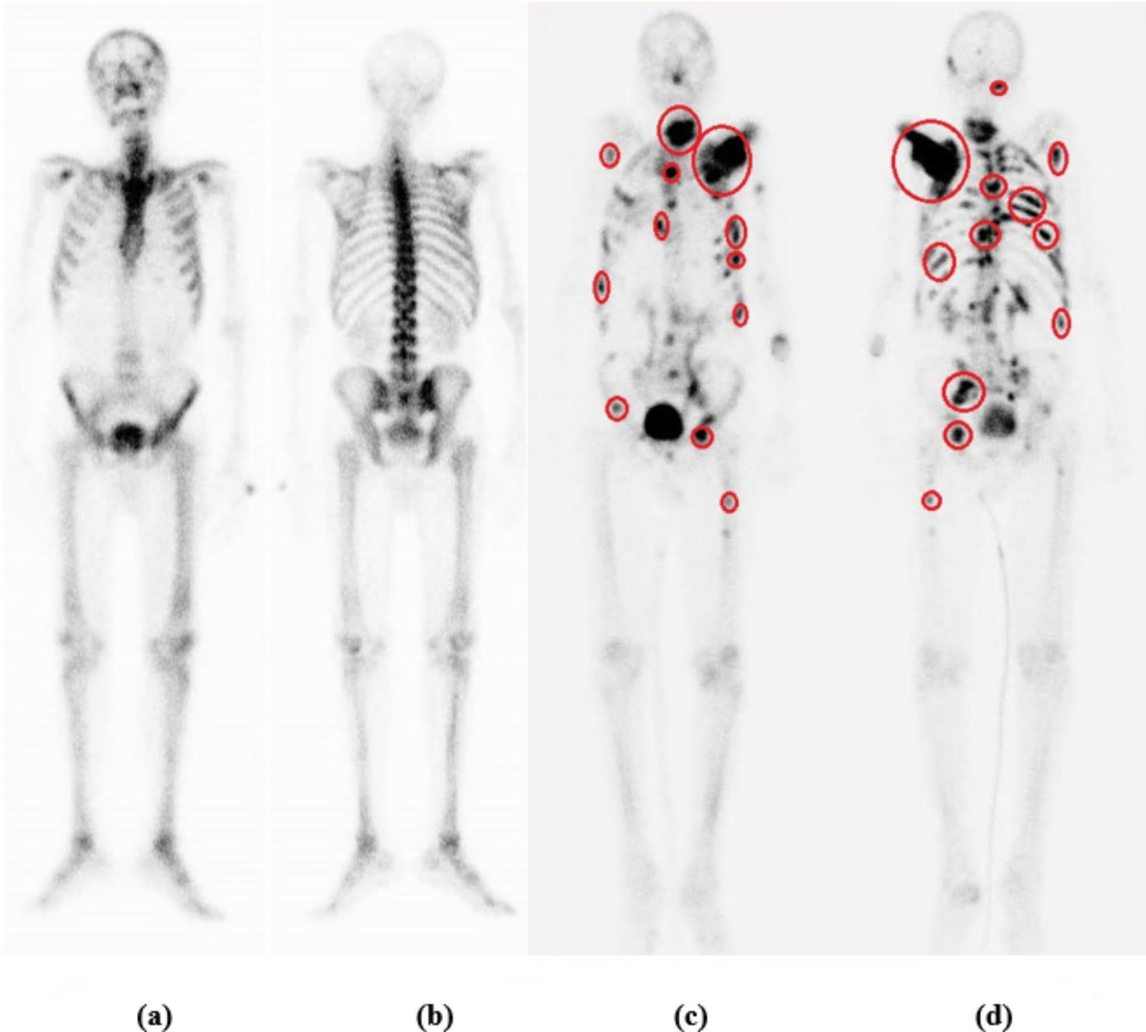
Skeletal scintigraphy.

In this investigation, images were gathered retrospectively from 2800 patients to assess the efficacy of our proposed algorithm, employing MobileViT for feature extraction. This dataset comprised 1400 abnormal and 1400 normal cases, encompassing both women and men aged between 3 to 90 years, who had previously undergone skeletal scintigraphy imaging for suspected bone metastatic investigation. The resulting images were two-dimensional, consisting of both anterior and posterior views, which were subsequently separated into distinct images. Figure 7 illustrates image samples from our dataset, where ‘a’ represents the anterior part of the normal scan image, ‘b’ depicts the posterior part of the normal scan image, ‘c’ shows the anterior part of the abnormal scan image, and ‘d’ displays the posterior part of the abnormal image. The study of this experiment adhered to the principles outlined in the Declaration of Helsinki and received approval from the Ethics Committee at Faculty of Science, Mansoura University (Code number: Sci-Phy-M-2021-79, issued on December 21, 2021). Informed consent from patients was waived by the Institutional Review Board (IRB) of Mansoura Faculty of Medicine due to the retrospective nature of the study.

#### Discussion of bone metastasis datasets

Table 12 provides a comprehensive summary of the outcomes, presenting various metrics for the objective function. The results underscore the promising performance of the proposed GOAOA algorithm. It claimed the top position in fitness values for both worst and best outcomes among all algorithms considered. While BPSO excelled in standard deviation results, the average of the objective function placed WOA in the first position, with GOAOA following closely in the second position. It is worth mentioning, the feature extraction model based on MobileViTv2 has a total of trainable 1,114,107 parameters when trained on this dataset resulting in an estimated total size of 70.34 MB. The average training time per epoch is approximately 15 s, and the inference time for the test set is around 2.4 s.

Moreover, the proposed GOAOA demonstrated exceptional levels of accuracy, sensitivity, and specificity, achieving the highest scores in these critical metrics. These results collectively emphasize the efficacy of GOAOA in managing real medical data associated with bone metastasis, positioning it as a promising algorithm for applications in this field.

**Table 12:** GOAOA performance with datasets on bone metastases.

Fitness function	BPSO	WOA	HHO	SSA	GO	AOA	GOAOA
Max	0.4423	0.2043	0.2091	0.2515	0.2825	0.2094	**0.2038**
Min	0.4423	0.1799	0.1927	0.2363	0.2707	0.1887	**0.1798**
Std	**0.000**	0.00803	0.00527	0.004973	0.003994	0.007031	0.0063
Avg	0.4423	**0.18873**	0.19832	0.24398	0.27701	0.20058	0.19465
Accuracy	0.7625	0.80540	0.8054	0.8018	0.7857	0.8107	**0.8268**
Sensitivity	0.7714	0.8714	0.8643	0.8357	0.8143	0.85	**0.8857**
Specificity	0.9286	0.9500	0.9524	0.9476	0.9405	0.9548	**0.9595**

Bold indicates the best value.

In summary, the results and explanation of the developed technique underscore its robust capacity to detect bone metastasis and effectively categorize UCI datasets. This dual proficiency emphasizes the versatility and promise of our approach, making it a valuable asset in medical diagnostics and broader data classification applications. Additionally, the performance of most algorithms is better on certain datasets than others since each one of those datasets is considered an optimization problem. Therefore, according to non-free launch theory “no one algorithm can solve all problems with the same performance”.

In addition, the proposed method offers distinct advantages in automating and streamlining the diagnostic process, thereby reducing the reliance on manual interpretation and improving efficiency. By integrating advanced techniques like Mobile Vision Transformer and Arithmetic Optimization Algorithm for feature extraction and selection, respectively, the model enhances accuracy, consistency, and scalability in analyzing large volumes of scans. However, challenges such as dependence on the quality of training data, interpretability of decisions, technical expertise requirements, and regulatory compliance need careful consideration to ensure successful integration into clinical practice and maximize its potential impact on patient care and diagnostic outcomes.

## Conclusion and future work

Metastatic bone disease remains a prevalent clinical concern and one of the most formidable diseases. Despite the fact that we used highly modern techniques and had the assistance of numerous experienced radiologists, early detection is still a pipe dream. So, we proposed a method by using the Mobile Vision Transformer model using two primary components, including ViT and lightweight CNN as feature extraction and a hybrid version of the Growth Optimizer (GO) and Arithmetic Optimization Algorithm (AOA) called GOAOA as feature selection to improve the accuracy of bone metastasis identification in bone scans via feature extraction and selection. The suggested method’s performance was evaluated using 2800 bone scan images, and the findings were compared to those of competing algorithms. The experimental findings demonstrate the efficacy of the GOAOA algorithm, which obtains excellent classification sensitivity, specificity, and accuracy. In the future, GOAOA will be used to tackle further issues in a variety of domains, including big data, sentiment analysis, smart cities, and other practical engineering issues.

## Data Availability

The data used in the first experiment are available at the UCI Machine Learning Repository (https://tinyurl.com/UCIDS ), while the data used in the second experiment (bone scan images) can be accessed upon reasonable request from the first author.
